# What makes community-based, multilevel physical activity promotion last? A systematic review with narrative synthesis on factors for sustainable implementation

**DOI:** 10.1177/17579139231186693

**Published:** 2023-08-04

**Authors:** N Helsper, L Dippon, L Birkholz, A Rütten, S Kohler, P Weber, K Pfeifer, J Semrau

**Affiliations:** Department of Sport Science and Sport, Friedrich-Alexander Universität Erlangen-Nürnberg, Gebbertstraße 123b, Erlangen 91058, Germany; Department of Sport Science and Sport, Friedrich-Alexander Universität Erlangen-Nürnberg, Gebbertstraße 123b, Erlangen 91058, Germany; Department of Sport Science and Sport, Friedrich-Alexander Universität Erlangen-Nürnberg, Gebbertstraße 123b, Erlangen 91058, Germany; Department of Sport Science and Sport, Friedrich-Alexander Universität Erlangen-Nürnberg, Gebbertstraße 123b, Erlangen 91058, Germany; Department of Sport Science and Sport, Friedrich-Alexander Universität Erlangen-Nürnberg, Gebbertstraße 123b, Erlangen 91058, Germany; Department of Sport Science and Sport, Friedrich-Alexander Universität Erlangen-Nürnberg, Gebbertstraße 123b, Erlangen 91058, Germany; Department of Sport Science and Sport, Friedrich-Alexander Universität Erlangen-Nürnberg, Gebbertstraße 123b, Erlangen 91058, Germany; Department of Sport Science and Sport, Friedrich-Alexander Universität Erlangen-Nürnberg, Gebbertstraße 123b, Erlangen 91058, Germany

**Keywords:** programme sustainability, health promotion, whole-system approach, multilevel physical activity promotion, community, systems change

## Abstract

**Aim:**

To follow the need for more research and strategies to enhance the knowledge of sustainable implementation, we examined cases of community-based, multilevel physical activity-related health promotion after initial funding has ceased and aimed to identify factors that influence their sustainable implementation.

**Methods:**

Five scientific databases (PubMed; Scopus; Ebsco Host with CINAHL, PsychInfo, and Sportdiscus; ProQuest and Web of Science) were systematically searched for relevant literature in December 2021. Three reviewers performed a title/abstract screening and independently screened the full texts of the remaining papers, followed by a quality assessment. A narrative synthesis method, including qualitative text analysis, was used to synthesise retrieved articles. As starting point, the framework of Schell et al. containing nine domains for sustainability capacity was used and new emerging themes were inductively added.

**Results:**

The search revealed 270 potentially eligible articles out of 27,652 hits. After the systematic review process, 14 studies were included. In the synthesis, 14 factors influencing the sustainablity of community-based, multilevel physical activity-related health promotion were identified of which six are new factors compared to Schell et al. In particular, our findings bring forth a novel understanding of the importance of the factors ‘Participation and Partnerships’, ‘Empowerment and Capacity Building’ and ‘Community Support’. A dynamic interplay and high connectedness between factors were visible.

**Conclusion:**

The identified factors can help establish a better understanding of sustainability processes within whole-system approaches intervening on multiple levels in the community with the aim of systems change. They are relevant for practitioners, researchers and policy makers alike. Future research should more closely examine based on further theoretical elaboration how an interplay between the factors can promote sustainability and which interdependencies are of particular importance in facilitating sustainable and equitable change.

## Introduction

Physical activity is well known for its versatile health-enhancing effects^1–3^ and its potential to reduce the economic burden resulting from physical inactivity.^4,5^ However, current global reports show that one in four adults (27.5 %)^6^ and three-quarters (81 %) of adolescents^7^ do not meet the recommendations for physical activity to enhance and protect their health. These data were collected before the COVID-19 pandemic began. A new study shows, that as of early 2022 worldwide step counts remain even significantly lower than their pre-pandemic baseline.^8^ Hence, the importance to promote physical activity has increased globally in recent years.

For physical activity promotion, the *Global Action Plan on Physical Activity 2018–2030*^9^ emphasises systems-based approaches and a focus on sustainable and equitable implementation. A discourse on sustainable implementation is currently of particular relevance due to impending funding shortages.^10^ These threaten the continuation as well as adaptation of existing efforts and can especially hinder the sustainable implementation of new comprehensive systems-based approaches. At the same time, the expectation to generate quality results remains high. The field of health promotion is caught in a dilemma: ‘to provide quality services that reduce disease burden and improve quality of life, while under budgetary constraints’ (p. 2).^11^ This creates a need for health promotion to be sustained, to ensure that the health benefits gained from the approaches do not terminate with its completion.^11^ Thus, for the efficient use of resources (e.g. financial, human and time) and to achieve a public health effect, it is crucial to increase the understanding of sustainable implementation in the real-world.^12^ Against this background, this review refers to sustainability as ‘to what extent an evidence-based intervention can deliver its intended benefits over an extended period of time after external support from the donor agency is terminated’ (p. 118)^13^ and follows thereby the *Glossary for Dissemination and Implementation Research in Health*.

One significant real-world setting for health promotion is the community since the Ottawa Charter in 1986.^14^ In this article, the community is defined as a geographic region (e.g. urban and rural municipalities and districts). A community-based approach can affect all social determinants of health^15^ and is hence recommended to reduce health inequities^15^ if vulnerable populations are particularly addressed.^16–18^ Therefore, approaches at the population level, which consider the whole system of a community and health equity issues, are promising for the sustainable and effective (i.e. achieving a public health effect) implementation of physical activity-related health promotion (PArHP).^9,19,20^

To improve the understanding of how to take the whole system of a community into account and how to initiate sustainable change within it, we draw on the multilevel model of Rütten and Gelius^21^ as a valuable theoretical basis. The multilevel model describes the interplay of structure and agency^22^ in health promotion and is connected to central claims of the Ottawa Charter.^14^ These are ‘build healthy public policy’, ‘create supportive environments’, ‘strengthen community actions’ and ‘develop personal skills’.^21^ When applying the multilevel model to community-based PArHP, four dimensions or ‘entry-lanes’ for community systems change arise: (1) policies promoting physical activity, (2) citizen engagement, (3) infrastructures promoting physical activity and (4) physical activity behaviour. In this review, community-based PArHP combining two or more of these four interplaying dimensions is considered as multilevel PArHP and has the potential to affect the whole community system.

Keeping the dynamics of community systems active is challenging. This is because a community system needs to adapt itself amid constantly changing contexts, such as staff turnovers or new funding policies and political priorities.^23–25^ Therefore, resilient and sustainable, meaning adaptive, systems are necessary.^24,26^ Besides this, the sustainability of multilevel PArHP plays an important role because community system changes take time to achieve.^27^ Hence, a public health impact is measurable only after a long period of time.^28^ In addition, a sustainable embedment of systems change approaches, such as multilevel PArHP, is also increasingly demanded by policies and funding agencies, as they can be considerably resource intensive.^29^

However, at present, research that specifically evaluates factors affecting the sustainable implementation of community-based multilevel PArHP is scarce even though its importance is emphasised by several studies and guidelines.^9,30^ So far, researched sustainability factors primarily relate to the organisational and delivery processes of small-scale health promotion programmes or interventions.^11,24^ Population-based approaches that aim at systems change in the community and intervene on multiple levels remain largely unknown.^24,28,31^ Factors that consider the complex intervening influences on sustainability of entire community systems are required^24,27,31^ as linear influences may no longer apply to multilevel approaches.^32^ There are several methodological reasons that contribute to this knowledge gap. First, varying understandings of sustainability, such as differing perspectives on the acceptability of changes and adaptations within the chosen approach. Second, inadequate funding for post-implementation evaluations. Third, difficulties in real-time and multilevel observation, posing challenges to data collection. And finally, the lack of validated measures to assess sustainability^33^ as well as systems changes (e.g. progress in policymaking).^12^ As whole communities are dynamic and complex systems involving many parties and interests,^34^ additional factors compared to existing frameworks are to be expected.

Schell et al.^35^ developed a well-recognised conceptual framework describing nine core domains that affect a programme’s capacity for sustainability. Their understanding of sustainability capacity ‘moves beyond the characteristics of the programme itself that might support its sustainability to include organisational and systems characteristics’ (p. 2).^35^ Since the initial publication of the 2013 framework, it has been shown that multilevel approaches are important for long-term impact in addressing complex issues such as physical inactivity.^24^ Therefore, in this article, we built on the findings by Schell et al.^35^ but further focus on factors affecting the sustainable implementation of multilevel PArHP in the community system.

In summary, to follow the expressed need for additional research and strategies to enhance the knowledge of sustainable implementation,^36^ we examine cases of community-based, multilevel PArHP after the initial funding has ceased and aim to identify the factors that influence their sustainable implementation.

## Methods

To answer the question ‘What are the factors for the sustainable implementation of community-based, multilevel physical activity promotion?’, we conducted a systematic review with narrative synthesis. A narrative synthesis following the guidelines by Popay et al.^37^ was selected because of its exploratory nature and the ability to integrate knowledge from diverse methodologies and approaches. After a systematic literature search,^38^ the review followed the steps for a narrative synthesis as proposed by Popay et al.:^37^ (1) developing a preliminary synthesis, (2) exploring relationships within and between studies and (3) assessing the robustness of the synthesis. The step ‘theory development’ was not conducted due to the exploratory research approach as described by other authors.^39^

### Systematic identification of relevant literature

To identify relevant literature, we combined search terms regarding the four topics: (1) physical activity, (2) system approach, (3) sustainability and (4) community (see Supplement 1). The search strategy was applied in five scientific databases (PubMed; Scopus; Ebsco Host with CINAHL, PsychInfo, and Sportdiscus; ProQuest and Web of Science) in December 2021, without any time restrictions. We only searched for papers in English and German language. No additional grey literature search was conducted, and no authors were contacted for information or references. The research team imported all results into the bibliographic management software programme Citavi 6.10 to organise the selection process and to remove duplicates automatically.

### Selection of the literature

Given the heterogeneous terms used in the literature, a broad search strategy was applied. A checklist with the inclusion and exclusion criteria (see Table 1) was first developed and discussed to ensure mutual understanding within the research team. Subsequently, LD, LB and NH crosschecked the title/abstract screening results and independently screened the full texts of the remaining papers. Differences in the full-text screening were discussed with a fourth person (JS) to clarify any discrepancies.

**Table 1:** Inclusion and exclusion criteria

Inclusion criteria
**Physical activity promotion must be one main objective**
**Community-based PArHP including the involvement of the municipal/district level (i.e. the administration)**
**Multilevel approach (i.e. combination of two or more of the four described dimensions by Rütten and Gelius** ^21^ **) applied to PArHP**
**Reporting of positive or negative factors influencing the sustainable/non-sustainable implementation of PArHP after the initial funding has ceased**
**Peer-reviewed publications: quantitative, qualitative, mixed-methods studies, reviews and reports**
Exclusion criteria
**Exercise therapy and rehabilitation interventions**
**Clinical settings**
**Only individual-based, behaviour-related measures (if they were not part of a multilevel approach)**
**Only one setting in the community involved (e.g., only school setting)**
**Description of sustainability planning and no retrospective report of sustainability after the initial funding has ceased**

PArHP, physical activity-related health promotion.

### Data extraction and analysis

For preliminary synthesis, the tabulation method was selected to extract the descriptive characteristics of the included studies. In addition, a qualitative text analysis^40^ was performed to identify the factors that positively or negatively influence the sustainable implementation of community-based, multilevel PArHP. As a starting point, the framework of Schell et al.^35^ containing nine domains of capacity for sustainability (‘Funding Stability’, ‘Political Support’, ‘Partnerships’, ‘Organizational Capacity’, ‘Program Adaptation’, ‘Program Evaluation’, ‘Communications’, ‘Public Health Impacts’ and ‘Strategic Planning’) was used, and new emerging themes were inductively added. For this, LB and NH first independently coded two included full texts and in a mutual discussion, a codebook was then developed and again tested on a third paper. Subsequently, LB and NH coded each half of the papers and then alternated to allow for inter-rater reliability. Discrepancies were again discussed with a third person (JS). For qualitative text analysis, the software programme MAXQDA 2020 was used. First-order constructs (data collected by the observer or researcher), as well as second-order constructs (statements of theory, causality or principle based on first-order data) were considered in the data analysis.^41^

The exploration of relationships between studies is described in the “Discussion” section.

### Quality appraisal and assessment of the robustness of the synthesis

LB and NH completed a quality appraisal of the studies using a pragmatic quality assessment checklist.^42^ With four questions, the checklist allows to assess whether the studies clearly describe (1) the research question, (2) the study design, (3) the method of participant selection and (4) the data collection and analysis methods.^42^ If studies provided sufficient information about two or more of these criteria, they were classified as adequately reported. Inadequately reported studies from the analysis were not excluded in the review. The conclusion includes a critical reflection on the synthesis process to check for the robustness of the synthesis.^37,43^

## Results

### Overview of the identified studies

**Figure 1:**
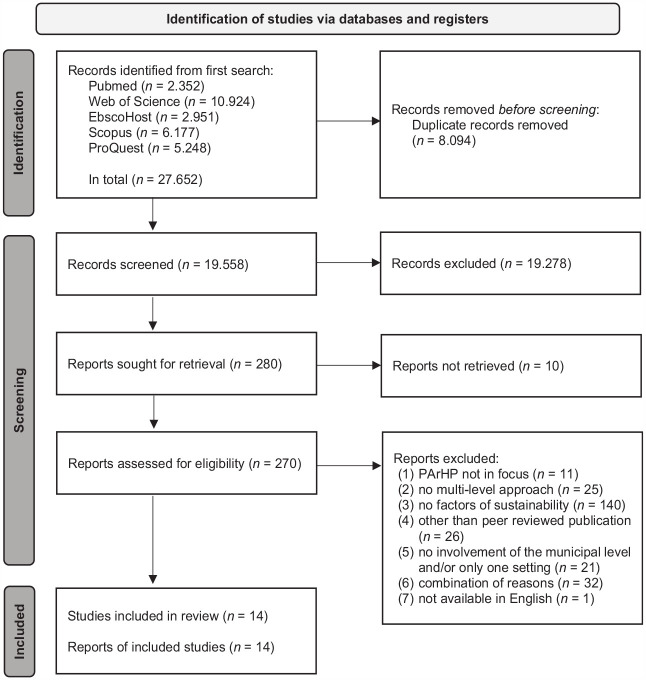
Preferred reporting items for systematic reviews and meta-analyses (PRISMA) 2020 flow diagram of the identified studies

A Preferred reporting items for systematic reviews and meta-analyses (PRISMA) 2020 flow diagram^38^ (see Figure 1) was used to present the results of the systematic literature search, which identified 14 studies (reports of included studies) with 14 different community-based, multilevel PArHP programmes (studies included in review). The earliest and latest publications were from 2009 to 2020, respectively. The programmes were located in North America (*n* = 6), South America (*n* = 4), Europe (*n* = 3) and Africa (*n* = 1). The general population, specifically adults, were mostly targeted. Regarding health equity, disadvantaged populations were particularly considered in nine programmes. In the included multilevel programmes, mostly, a combination of intersectoral partnership building, development of new offers and educational classes and events was conducted. Policies, environmental infrastructures and new personnel resources were implemented less often, even though they are considered more sustainable^44–46^ and successful in improving health equity.^30^ The multilevel programmes varied in duration. Some were active for almost 10 to 20 years at the point of evaluation,^45,47–51^ whereas others just transitioned from the initial impetus to new forms of funding and organisation.^46,52–56^ The used sustainability concepts differed between studies but not on a large scale. For instance, five studies define sustainability similar to this review. Others emphasise the creation of lasting changes and increased capacity in the community, as well as the transfer to new settings and ongoing adaptation (see Supplement 2). As a methodology, the majority selected a (multiple) case study design. In the sustainability assessment, the studies collected data mostly through interviews and documents (e.g. systems and policy change tracking forms, grantee/project annual reports and community action plans) for further qualitative, quantitative and cross-case analysis. All studies examined the factors influencing the sustainability of the respective programmes. Information regarding maintained PA effects were scarcely measured, if only in terms of reach such as by number of participants^47^ or awareness of the programme.^52^ Reported factors that influenced the sustainability of the community-based multilevel PArHP programmes were coded. The results are described in the next chapter.

Further descriptive characteristics of the 14 studies are displayed in Supplement 2, including all sustainability concepts, the methods used for sustainability assessment and short programme overviews. The quality appraisal classified 12 out of 14 studies as adequately reported.

### Factors for the sustainable implementation of community-based, multilevel physical activity-related health promotion

**Figure 2:**
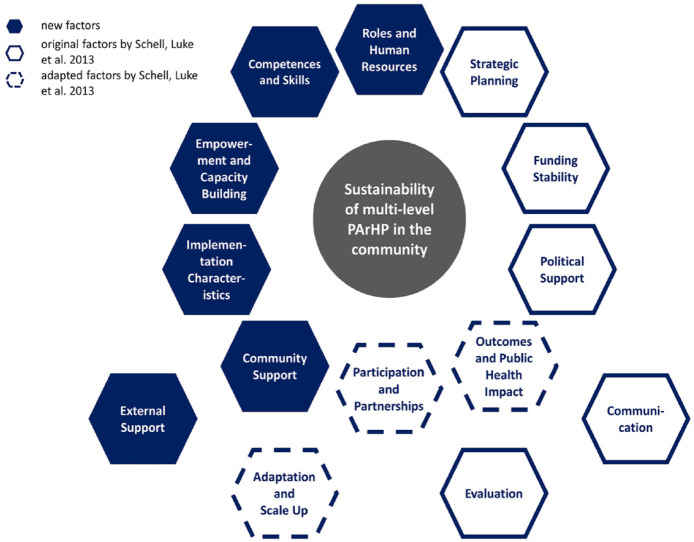
Fourteen sustainability factors for community-based, multilevel, physical activity–related health promotion (PArHP)

**Figure 3:**
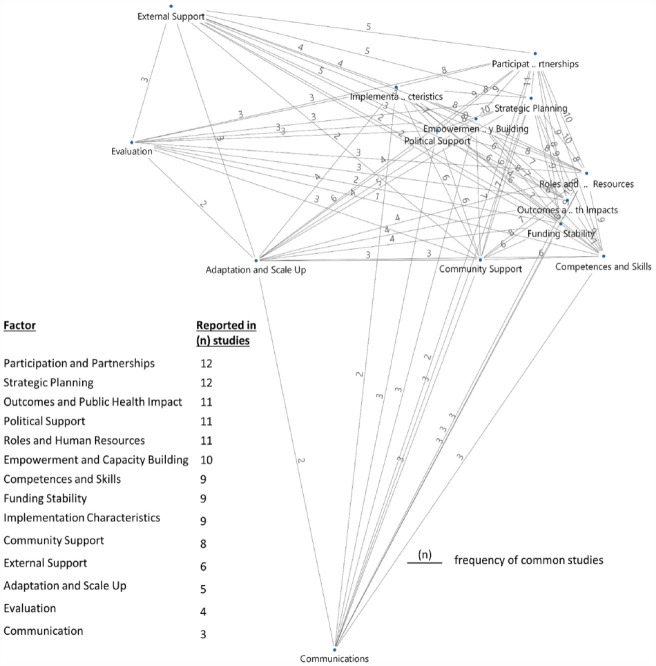
‘Participation and Partnerships’ and ‘Strategic Planning’ are the most commonly reported sustainability factors

Through qualitative text analysis, six new factors were found in addition to the factors of Schell et al.^35^ for sustainability capacity (see Figure 2). Furthermore, three factors by Schell et al.^35^ have broadened in their scope: (1) ‘Program Adaptation’ has been adapted to ‘Adaptation and Scale Up’, (2) ‘Partnerships’ has been adapted to ‘Participation and Partnerships’, (3) ‘Public Health Impacts’ has been adapted to ‘Outcomes and Public Health Impacts’. Moreover, the original factor ‘Organizational Capacity’ by Schell et al.^35^ has been split and is now reflected in detail in several new factors such as in ‘Roles and Human Resources’ and ‘Competences and Skills’. In summary, 14 factors that can influence the sustainable implementation of community-based, multilevel PArHP were identified. When examining the appearance of the identified factors, apparently, a combination of 10 factors were most commonly mentioned within the same study (see Figure 3). These 10 factors were also most often reported. Regarding the relationships between factors, no chronological sequence was described in the studies; however, a dynamic interplay and high connectedness were visible. Therefore, we decided to arrange the most common factors in a circle and add the remaining four underneath (see Figure 2). Nevertheless, this does not indicate that these four factors are less important. The following presents descriptions of all identified factors based on the results of the qualitative text analysis and contains core ideas and perspectives.

#### Roles and human resources

For the sustainable implementation of PArHP it needs people with diverse roles and sufficient time resources. Moreover, coordinators in paid and in best case permanent positions are crucial for the institutionalisation and the creation of sustainable changes.^44,50,54,56^ In addition, qualified champions who advocate at various levels and sectors^47,54^ sufficient volunteers^46,53^ and qualified exercise trainers are highlighted.

#### Strategic planning

The studies underline that building a common shared vision,^46,56^ setting priorities,^46,56^ anticipatory planning of resources,^47,52,53,57^ starting with small activities followed by subsequent scale-up,^47,56^ and considering sustainability from the beginning on^46^ are important aspects. The integration of sustainability processes with the implementation of activities in multiple levels^53,56^ is particularly mentioned since some case studies show that a sole focus on activities, such as PA classes, leads to difficulties in the broader sustainable embedment of PArHP in the community.^53^ In addition, the strategic application of a social–ecological approach is one used key concept.^52,55^ Furthermore, selecting partners with strong potential (i.e. those that have resources, e.g. time and finances, and knowledge),^54^ as well as a systematic participatory process that keeps the partners engaged,^46,51^ are pointed out.

#### Funding stability

We identified the lack of financial resources after the initial funding has ceased as one main barrier for sustainability.^45,47,50,52–54,57^ In this context, subsidy schemes have a great influence. Spending restrictions and grant requirements can be viewed as problematic and beyond the control of the community.^53^ In addition, communities requiring ongoing efforts to raise funds are forced to increase programme fees,^57^ which act contrary to health equity efforts. Possible solutions are the inclusion of subsidies from other sectors, such as public housing,^54^ and the combination of diverse funding sources.^39,50^ On the political level, the provision of implementation budgets along with new policies^47^ is also underlined to allow the translation of policies into practice.

#### Political support

The support of decision-makers on various levels^51,54^ (e.g. in national and regional politics, organisations and administrative departments) and their priorities^44,45,48^ influence the likelihood of sustainability. Their actions affect budgets and the approval of PArHP policies during legislative periods.^45^ A combination of policies and allocated budgets, as well as PArHP policies and joint actions across several sectors and levels, may have a positive effect.^47–49,57^

#### Outcomes and public health impact

To justify the use of resources, stakeholders often have to legitimate their actions. Thus, positive outcomes indicating an effective implementation are important in achieving a sustainable community systems change.^45,56^ Here, environmental and policy changes are considered more sustainable than programme-based activities.^35,37^ Only if actions reach the population, the viability of the community system for PArHP is strengthened, which, in turn, positively influences sustainability and the population’s health in the long run.^50,57^ ‘Policy and systems changes will not be effective if the culture does not support the change’ (p. 89)^46^ is important to consider. Therefore, values should be integrated into collaborations and new efforts for change.

#### Implementation characteristics

The studies demonstrate that the way multilevel PArHP is implemented has an effect on sustainability. For example, we found that compatibility with needs,^52^ public relevance,^53,55^ tailoring to the (social and cultural) context,^45,46,51^ an equity lens,^56^ as well as integration of local structures,^48,57^ play an integral role. Moreover, Herens et al.^57^ reported the linkage of related activities as beneficial, such as health education with exercise classes.

#### Community support

Many PArHP efforts rely on community support, which is considered as the engagement of the population, such as volunteers and advocates. When volunteers are missing, the sustainability of the intervention can be endangered.^53^ Community support can also be a key for maintaining/expanding services. For instance, Castillo et al.^47^ described a case in which citizens protested after a co-funding agreement was terminated. This protest impelled the authorities to hire new personnel and to continue the programme. Continued community support can also make it difficult for any policymaker to risk making an unpopular decision concerning the programme.^48^

#### Participation and partnerships

Sound social structures combined with trustful relationships are essential for long-term collaborations, resource allocations and partnerships.^50,53,55–57^ More importantly, the partners can rely on each other and use a shared leadership concept that does not depend on single persons/champions alone.^49,56^ Diverse and multisectoral partnerships are necessary on several levels.^44,45,51^ The involvement of the municipal and/or governmental level seems to be particularly relevant.^44,50,51,55,57^ Regarding participation, we identified that the equal engagement of population groups during planning and implementation (e.g. leading PA programmes) positively influences the sustainable implementation of PArHP.^46,49,51^

#### Empowerment and capacity building

‘The view supported by our work is that empowerment at multiple levels seeks to ignite changes that, in turn, contribute to sustained impact of outcomes’ (p. 7).^55^

On the individual level, strategies for empowerment and capacity building can be the development of skills,^46,47,53^ as well as taking leadership,^49^ through, for example, active participation in the implementation of activities. This affects stakeholders working in the community, as well as engaged population groups. On a community level, the setup of ownership as well as an increase in the feeling of control, for example in regards to financial resources, build capacity for sustainability.^46,49,52,57^

#### Competences and skills

We identified good communication and advocacy skills as especially important^49,50,57^ next to identifying and leveraging resources.^44,53,56,57^ In addition, the resilience towards setbacks and the continued commitment and motivation of actors in systems-changing processes may be substantial.^49,53,57^ Furthermore, the quality of ‘physical activity instructors who are in direct contact with the participating community’ (p.8)^45^ matters for the maintenance and reach of programmes.

#### Evaluation

Evaluation can contribute to the continued implementation of PArHP through, for example, process monitoring, which supports quality control and credibility,^52^ as well as the provision of guidelines for future scale-up.^49^ Furthermore, Díaz Del Castillo et al.^48^ indicated that an interdisciplinary evaluation group of researchers, practitioners, stakeholders, programme coordinators, and community leaders helps to provide public health evidence on the effectiveness of activities, ‘which in turn could help policy development, dissemination, and sustainability’ (p. 358).^48^

#### External support

Collaborations with research institutions are the most common external support reported. A renowned institution can help boost the credibility of an intervention,^49^ and experiences can be exchanged, for example, regarding how the population can be involved.^50^ Furthermore, an interdisciplinary team approach can address complex scientific and societal problems, such as population-based PArHP, more holistically.^55^ We also identified working materials, such as manuals or websites,^52^ and an (intermediate) support structure that provides central consultation and training^51,52^ as relevant.

#### Adaptation and scale up

The ability to respond to contextual dynamics and changing priorities is essential and emphasises that sustainability in itself is a dynamic process.^23,52^ Díaz Del Castillo et al.^47^ also described this as a need and willingness to be flexible, for instance, by adapting to people’s preferences and funding changes. The transfer to new settings and communities can foster sustainable systems for multilevel PArHP as it broadens the scope and increases the reach for public health impacts.^51^ Continuous adaptations are essential for a strong and active community system.^47^

#### Communication

Communication actively takes place in partnerships through sharing expectations and values behind participation and, for instance, in the direct approach of the population through word-of-mouth.^57^ (Social) media can also share information about activities including good news and stories to increase acceptance, support, and reach of the PArHP approach.^47,53,57^ Advocacy, especially by decision-makers, that underlines the importance and the added value of PArHP for the community plays a key role in building momentum.^47,53^

## Discussion

This review identified 14 factors influencing the sustainable implementation of community-based, multilevel PArHP after the initial funding has ceased. The factors result from a complex systems perspective^28^ and reflect the necessary evolution of the whole community to achieve sustainable PArHP. This may be a reason why we found more factors than Schell et al.^35^ who examined sustainability capacity of public health programmes in general and less within the community system. The 14 factors add new insights to community systems change approaches and support findings of previous key papers regarding health promotion programme sustainability.^29,31,58^

In particular, our findings bring forth a novel understanding of the importance of the factors ‘Participation and Partnerships’, ‘Empowerment and Capacity Building’ and ‘Community Support’. These emphasise how continued participation of the population can result in enhanced ownership, capacity^46–51,59^ and in turn, sustainability.^58,60^ The concepts of participation and empowerment have been substantial for health promotion for a long time.^59,61^ However, it has been scarcely discussed in terms of sustainable community systems change. Through choosing a participatory action research approach, some included studies shed extra light on factors related to the involvement of the community. ‘External Support’ is another newly established factor that stands out. It has already been studied in terms of scaling-up processes ^62,63^ where similar functions, such as technical assistance or evaluation support, have been described.^63^

A crucial insight from our analysis is that the included studies did not describe any links to chronological sequence or the point of time when each sustainability factor should be addressed. One explanation may be that as community systems are complex, they do not behave in a linear fashion.^64,65^ For actions to take effect, they must first reach a tipping point and then pass through a following transition phase.^64^ Taking this into account, the factors’ intensity, pace (e.g. how intense and how often a factor is addressed) and interplay may have a more important role than sequence. Similar to Myron’s maxim ‘Start anywhere, follow it everywhere’ (p. 23),^66^ any change can lead to the initiation of further changes as ripple effects^67^ and are worth following. Fittingly, we found sustainability in accordance with Chambers et al.^23^ to be a dynamic and iterative process that should be addressed from the beginning of implementation and onwards.^46,53,56^ This dynamic process involves ‘continued learning and problem solving’ (p. 1),^23^ ongoing adaptation between the PArHP approach and the multilevel community system, as well as ‘ongoing improvement, as opposed to diminishing outcomes over time’ (p. 1).^23^ It is significant that a dynamic interplay and high connectedness between the factors become visible here.

In this context and expanding upon the existing body of literature, this review highlights the importance of time for sustainability. First, sustainability happens within a certain passage of time (e.g. dynamic interplay between sustainability factors). Second, sustainability is influenced by factors that occur because of time. For example, at the current time long-term funding and longitudinal research face many competing societal challenges (e.g. the long-term effects of the COVID-19 pandemic, climate emergency, humanitarian and refugee crises).^68^ These require rapid reactions tending to increase a bias that prioritises short-term issues over longer-term focus.^69^ Moreover, the understanding of time has been broadened in the 14 identified factors of this review. In the 2013 framework of Schell et al., time can be associated with ‘Organizational Capacity’. In our factors, time is directly reflected in ‘Roles and Human Resources’ and ‘Strategic Planning’ underlining the growing economic importance of time for sustainable multilevel PArHP in the community. Although sufficient time and resources are needed, PArHP has the potential to reduce many non-communicable diseases,^3^ can decrease the economic burden resulting from physical inactivity,^4^ and is an interdisciplinary topic with potential synergies, for example, in the areas of climate protection^70^ and social integration.^71^ Therefore, PArHP is highly relevant for practitioners, policymakers and researchers in creating healthy and sustainable communities now and in the future.

Especially relevant for practitioners is that the 14 factors are strengthened by their high practical applicability. In sustainability research, the integration of scientific knowledge with practice-based knowledge has been repeatedly emphasised.^23,72–74^ A high degree of overlap exists between the scientifically generated factors in this review (scientific knowledge) and practice-based key components for effective and sustainable community-based physical activity promotion (practice-based knowledge).^34^ The key components were co-produced with stakeholders from practice, policy and research in two nationwide workshops in Germany in 2018/19.^34^ Differences between the scientific knowledge and the practice-based knowledge exist regarding ‘Evaluation’, ‘Adaptation and Scale up’, ‘External Support’, as well as ‘Outcomes and Public Health Impact’. These named factors were only identified in this review. ‘Infrastructural Resources’ (e.g. available bike paths, gyms and swimming pools) on the other hand, were only stated by the stakeholders. An explanation for the differences may be the stakeholders’ focus on rather rapid implementation than long-term systems-changing processes. This can also fit to the experiences made in the included case studies. They describe keeping balance between systems-oriented sustainability processes (e.g. coordination of partnerships, gaining political support) and physical activity outcomes and impacts (e.g. maintaining participation in physical activity classes) as challenging. Reasons for this can be long-lasting processes with considerable efforts and resources across sectors and political levels within funding periods that are too short.^33,35,36,39^

### Critical appraisal of the synthesis process and limitations

This review is not without limitations, and a critical appraisal of the synthesis process is needed to interpret the results in accordance with the quality of the integrated evidence. The first limitation regarding the sample of articles concerns the different concepts used for sustainability in the included studies. This consequently resulted in varying methods in the assessment and documentation of sustainability factors (e.g. regarding selection of interviewees, amount of data and survey method). Second, the studies assessed the factors for sustainability at different points of time. While some interventions have already been institutionalised, others were just in the transition from initial to new funding sources. This could have led to different emphases in interviews and reports by study participants. The third limitation relates to the lack of information about the community contexts and the applied multilevel approaches. Authors were not contacted for additional information that can improve the synthesis. Fourth, to deepen the knowledge regarding sustainability and changes over time, assessments should be done over several years rather than at a single point of time. However, this is only possible if funding institutions and policies adapt to the demand for longer-term budget allocations in cooperation with research and practice.

There are also significant strengths associated with this review and the including narrative synthesis process. First of all, the included studies chose primarily exploratory case study designs. The goal of case studies is to expand and generalise theories and not to extrapolate probabilities.^75^ Community change processes are considerably unique and complex that randomised controlled trials^76^ and replications may not be feasible.^27^ Therefore, the identified case studies are a valuable opportunity to learn from practical experiences in a scientific way. Furthermore, the narrative synthesis allows the inclusion of studies with different concepts and methodologies. Thus, the synthesis unites a wide range of insights strengthening the 14 sustainability factors. Collectively, the included studies exemplify strong models of sustainability. Because sustained implementation of the multilevel PArHP approach was achieved in the majority of studies, even after the initial funding ended, with some reporting partial sustainment or transitioning to new financial sources, while only one study did not achieve sustainment. The analysis of both successful case studies in terms of good practice as well as those that were not successful allows for a more comprehensive understanding of the underlying factors of sustainability. Moreover, the included studies were published across several countries in North (*n* = 6) and South America (*n* = 4), as well as Europe (*n* = 3) and Africa (*n* *=* 1). However, the applicability of the synthesis findings in communities located in other countries with different cultures may be reduced even though different types of communities (e.g. rural, urban, deprived and non-deprived) were considered. Finally, despite the broad search strategy, only 14 studies were eligible. On the one hand, this is a result of stringent inclusion and exclusion criteria. On the other hand, not many studies are present that have examined the sustainability of multilevel PArHP in the community after the initial funding has ceased, yet. This strength underlines the novelty and need for more research in this field especially in hindsight with the benefit of the current review and its identification of hitherto overlooked, but influential, factors.

### Directions for future research

To initiate community systems change, Rütten and Gelius^21^ emphasised taking action on different levels (i.e. in the four dimensions of their multilevel model: (1) policies promoting physical activity, (2) citizen engagement, (3) infrastructures promoting physical activity and (4) physical activity behaviour) and the interplay between levels as crucial. Thus, for PArHP improvement, future research should examine more closely how the interplay between levels^21^ can facilitate sustainable and equitable change. For theoretical elaboration, future research should also explore where the 14 sustainability factors are located within the multilevel model.^21^ Research is additionally necessary regarding when and which factors or combinations of factors play a role and how they influence each other continuously. Furthermore, given the conceptual variability in the included studies, the 14 factors should be further developed and explored in other areas of health promotion to understand how and whether they may be generalised. In addition, research is needed in more countries to broaden the knowledge of sustainable and equitable community-based multilevel PArHP in diverse contexts.

### Directions for practice

As processes become more complex, ‘Competences and Skills’ of practitioners (e.g. PA and health coordinators in organisations or administrations, exercise trainers and urban planners), researchers and policymakers (community, regional and national level) must also develop to foster the implementation of multilevel PArHP on a lasting basis. This entails learning new skills and ways-of-working within time-pressured contexts. For instance, the facilitation of intersectoral partnerships, advocacy work, the conduction of participatory approaches and particularly the ability to understand systems across sectors and levels are demanded. Moreover, in a constantly changing environment processes still need to be effective, meaning that they have to adapt and be learned rapidly. As such, the COVID-19 pandemic is a good example for how processes had to adapt quickly to serve new needs and legal conditions across levels. Therefore, practitioners, researchers and policymakers should mutually investigate how systems-based knowledge (e.g. implementation of multilevel approaches and systems mapping as part of community assessments) can be integrated into ongoing practices and training of people working in the field of PArHP. In addition, they should work jointly to continuously discuss the emerging needs of community practitioners and how to address them. Not one person alone can achieve change in a community system. Hence, to consider all 14 factors, sound partnerships across sectors and levels, interdisciplinary teams as well as the participation of the population are necessary.

## Conclusion

In this review, 14 factors that can influence the sustainable implementation of community-based, multilevel PArHPs have been identified. The studies examined sustainability after the initial funding has ceased. These factors provide a comprehensive overview of the broad effort needed to sustainably and equitably shape a community system and are relevant for practitioners, researchers and policymakers alike. Between the factors, a dynamic interplay and high connectedness exists. Fittingly, we found sustainability to be a dynamic process, which should be addressed from the beginning of implementation onward. In summary, the 14 factors can help establish an improved understanding of sustainability processes within whole-system approaches that intervene on multiple levels in the community and aim for systems change. The likelihood of successfully transferring the factors to other areas of health promotion than physical activity is high, as a large overlap exists between our factors and the ones by Schell et al.^35^

Future research should examine more closely and based on further theoretical elaboration how the interplay between factors can promote sustainability and which interdependencies are of particular importance in facilitating sustainable and equitable change.

## Supplemental Material

sj-docx-1-rsh-10.1177_17579139231186693 – Supplemental material for What makes community-based, multilevel physical activity promotion last? A systematic review with narrative synthesis on factors for sustainable implementation Supplemental material, sj-docx-1-rsh-10.1177_17579139231186693 for What makes community-based, multilevel physical activity promotion last? A systematic review with narrative synthesis on factors for sustainable implementation by N Helsper, L Dippon, L Birkholz, A Rütten, S Kohler, P Weber, K Pfeifer and J Semrau in Perspectives in Public Health

sj-docx-2-rsh-10.1177_17579139231186693 – Supplemental material for What makes community-based, multilevel physical activity promotion last? A systematic review with narrative synthesis on factors for sustainable implementation Supplemental material, sj-docx-2-rsh-10.1177_17579139231186693 for What makes community-based, multilevel physical activity promotion last? A systematic review with narrative synthesis on factors for sustainable implementation by N Helsper, L Dippon, L Birkholz, A Rütten, S Kohler, P Weber, K Pfeifer and J Semrau in Perspectives in Public Health
