# Supplementary material for: What makes community-based, multilevel physical activity promotion last? A systematic review with narrative synthesis on factors for sustainable implementation
Source: Perspect Public Health. 2023 Aug 4;145(5):260–71. doi: 10.1177/17579139231186693 (PMC12457715; doi:10.1177/17579139231186693)
Supplement: sj-docx-1-rsh-10.1177_17579139231186693 – Supplemental material for What makes community-based, multilevel physical activity promotion last? A systematic review with narrative synthesis on factors for sustainable implementation [file sj-docx-1-rsh-10.1177_17579139231186693.docx]

**Supplement 1**: *Search Strategy*

|  | **„AND“** | | | |
| --- | --- | --- | --- | --- |
| **„OR“** | *Physical Activity* | *System Approach* | *Sustainability* | *Community* |
|  | physical activity | campaign | sustain* | county |
|  | physical activities | intervention | maint* | city |
|  | bike | program* | continuation | cities |
|  | bicycle* | approach | "capacity building" | municipal* |
|  | cycling | policy | institutionali* | urban |
|  | bicycling | policy | scale-up | town |
|  | walking | initiative | scaling-up | suburb |
|  | walking | "whole system" | scalability | rural |
|  | "active transport" | "whole of system" |  | "local authority" |
|  | sedentary | "systems approach" |  | community |
|  | "active living" | "built environment" |  | neighborhood |
|  |  | built environment |  | neighbourhood |
|  |  | "joined up" |  |  |
|  |  | "cross sector" |  |  |
