# Supplementary material for: What makes community-based, multilevel physical activity promotion last? A systematic review with narrative synthesis on factors for sustainable implementation
Source: Perspect Public Health. 2023 Aug 4;145(5):260–71. doi: 10.1177/17579139231186693 (PMC12457715; doi:10.1177/17579139231186693)
Supplement: sj-docx-2-rsh-10.1177_17579139231186693 – Supplemental material for What makes community-based, multilevel physical activity promotion last? A systematic review with narrative synthesis on factors for sustainable implementation [file sj-docx-2-rsh-10.1177_17579139231186693.docx]

**Supplement 2**: *Descriptive study characteristics*

| *Study no. #* | *Authors* | *Year/*  *Country* | *Methodology* | *Setting/ Population* | *Multi-level PArHP* | *Sustainability* |
| --- | --- | --- | --- | --- | --- | --- |
| #1 | Van Acker R, De Bourdeaudhuij I, De Cocker K, Klesges LM,  Willem A and  Cardon G^1^ | 2012/  Belgium | longitudinal study | community/ adult  population | "10,000 Steps" is a whole-community project with a socio-ecological approach to promote physical activity (PA) in adults, consists of individual, organisational, community and policy components, activities include e.g. posters, street signs, campaigns, networks | **concept** extent to which an evidence-based health program can deliver its intended benefits over an extended period of time after external support is terminated **assessment** RE-AIM framework, survey of 24 public services which adopted the project, two and a half years after project dissemination sustained implementation: proportion of organisations that continued via online survey  institutionalization: LoIn scales,  maintained PA effects:  comparison of citizens aware (reached citizens) and those not aware (citizens not reached) via telephone-administered long version of the IPAQ  **sustained implementation**  50% (12 of 24) of the organisations reported continuation almost one and a half year after the funding for dissemination ended  **maintained PA effects**  the group of citizens aware of ‘10,000 Steps’ reported significantly higher leisure time and household PA levels than those not aware |
| #2 | Berg BK^2^ | 2015/  USA  (Texas) | multiple case study | community/ whole-population | in 2008 the Texas State Legislature appropriated grants over two-year intervals for local "mayor’s fitness councils" to formulate and implement wellness and fitness programs across the state, activities in the communities included e.g. fitness expo, new physical activity opportunities, walking School Bus, dissemination of mini-grants | **concept** the continuation of activities after the initial impetus and resources that created a programme have subsided, organisational-level and community-level sustainability **assessment** three communities with different sizes, histories and capacities; document analysis, interviews, and site visits  **sustained implementation**  in 2 communities the programme was not sustainable on the community and the organisational-level, in 1 community the programme continued (organisational-level) but was threatened by a lack of partnerships (indicated unsustainable on community-level)  **maintained PA effects**  N.A. |
| #3 | Cheadle A,  Egger R, LoGerfo JP, Schwartz S and Harris JR^3^ | 2010/  USA  (Washington) | case study | community/ disadvantaged older adults | “Southeast Seattle Senior Physical Activity Network” (SESPAN), community-organising strategy to increase physical activity opportunities and reduce barriers among low-income, multicultural older adults, networking to: (i) make connections between two or more community organisations to create new senior physical activity programs; and (ii) build coalitions of community groups and organisations to assist in making larger scale environmental and policy changes to increase senior physical activity | **concept** sustainable changes are those that can be supported by organisational or other community resources in an ongoing way, sustainability after initial grant or other start-up funding ends  **assessment** evaluation of findings and lessons learned during implementation through an uncontrolled prospective design, data collected through program logs, counts of program participation, interviews, regular debriefing sessions with the SESPAN organiser  **sustained implementation**  several activities continued e.g., annual PA events, health coalition meetings, small-scale changes in the built environment  **maintained PA effects**  N.A. |
| #4 | Díaz del Castillo A, Sarmiento OL, Reis RS and Brownson RC^4^ | 2011/  Colombia, Brazil | two case studies | community/ adult  population | multi-sectoral initiatives to overcome inequalities and provide quality of life developed by governments 1) “Ciclovía (Recreovía)”: streets in Bogotá are closed temporarily to provide residents exclusive access for recreation and sports, complementary program of free PA classes in public spaces, temporary modules located next to the Ciclovía e.g. provision of counselling by health personnel and free public services 2) “CuritibAtiva”: city-wide program that promotes, directs, and evaluates PA in sports and leisure centres, parks, squares, and schools, activities are e.g. PA classes, Night Bikers, walking circuit | **concept** long-term program implementation **assessment** analysis of historical events that led to the programs’ origination and consolidation, identification of policies and factors that may have influenced their development and sustainability through a literature review (e.g. documents) and semi-structured interviews  **sustained implementation**  1) Ciclovía (Recreovía) has been active since 1974  2) CuritibAtiva has been active since 1998  **maintained PA effects**  N.A. |
| #5 | Díaz del Castillo A, González SA, Ríos AP,  Páez DC,  Torres A,  Díaz MP, Pratt M and Sarmiento OL^5^ | 2017/  Colombia | two case studies | community/ adult  population | “Ciclovía Recreovía” and “HEVS“ address access inequalities to PA through behavioural-social interventions and multi-sectoral collaborations; free, regular, and enjoyable PA classes at public spaces led by trained instructors 1) “HEVS”: monthly informational/educational outreach activities in community settings, schools, health services, and work sites.  2) “Recreovía”: daily PA classes and road closures | **concept** Sustainability is defined as a dynamic set of processes that allow for the continued delivery of a program's activities and policies in an identifiable form even if modified **assessment** examination of challenges and strategies related to the sustainability and scaling up by using a mixed-method design consisting of interviews and review of documents  **sustained implementation**  1) HEVS has been active since 2003  2) Recreovía has been active since 1995 after modernisation, original start was in 1974  **maintained PA effects**  1) HEVS: N.A.  2) Recreovía: number of participants decreased from 1.017.000 (2001) to 517.000 (2015) due to missing information |
| #6 | Draper CE, Kolbe-Alexander TL and  Lambert EV^6^ | 2009/  South Africa | cross-sectional study | community/ primary  school learners, adults and senior adults | “The Community Health Intervention Programmes” (CHIPs) are a physical activity-based health promotion programmes providing opportunities for health-enhancing, enjoyable physical activity within disadvantaged communities, they comprise five programmes based on a life course approach, which draw on principles of social cognitive theory, each programme includes a leader training | **concept** CHIPs branches that require very little follow-up and support from the CHIPs management team were regarded as more sustainable, whereas those branches that require much time and attention from CHIPs management were considered to be less sustainable **assessment** qualitative retrospective evaluation after 9 years of barriers and success factors regarding the implementation, naturalistic observation of CHIPS branches, structured interviews, focus groups at meeting venues, open-ended questionnaires, workshop with staff  **sustained implementation**  at the point of evaluation CHIPs have been operating for 9 years (start in 1997)  **maintained PA effects**  N.A. |
| #7 | Haggis C,  Sims-Gould J, Winters M,  Gutteridge K and McKay HA^7^ | 2013/  Canada | two case studies, literature review | school/  children and youth;  community/ adults over 65 years | both projects follow the participatory action research and social ecological approach 1) "Action Schools! BC": province-wide, physical activity focused, whole school health promotion model  2) "Active Streets Active People": project to identify links between the built environment and older adult physical activity and health | **concept** sustained impact defined as the implementation of an evidence-based program or policy that has lasting influence on health promoting behaviour **assessment** comparison of a critical evaluation of experiences and process evaluation data with findings from a literature review  **sustained implementation**  1) Action Schools! BC: activities have been continued in 1455 schools  2) Active Streets Active People: still under evaluation (2012-2014)  **maintained PA effects**  N.A. |
| #8 | Herbert-Maul A, Abu-Omar K, Frahsa A, Streber A and Reimers AK^8^ | 2020/  Germany | multiple case study  (17 sites) | community/women in difficult life situations | "BIG" aims at comprehensive and sustainable physical activity and health promotion among socially disadvantaged women, it empowers women through a participatory and asset-based approach, established coordinators organise the implementation, activities include e.g., aerobic fitness, Zumba, Nordic walking, and women-only indoor-pool hours | **concept** long-term implementation with maintenance of program activities, transfer to other contexts (new communities) **assessment** examination of the mechanisms of successful transfer and sustainability strategies: assessment of the years that BIG was maintained at the sites, reasons given by sites for discontinuation (contact by phone, reflection workshop, project documentation), each site’s costs regarding the transfer of BIG  **sustained implementation**  BIG has been active since 2005, 17 sites implemented BIG, 7 sites have been continuing BIG  **maintained PA effects**  N.A. |
| #9 | Herens M, Wagemakers A, Vaandrager L, van Ophem J and Koelen M^9^ | 2017/  Netherlands | multiple case-study | community/  disadvantaged populations | "Communities on the Move" was developed and disseminated in line with national policy objectives by the Netherlands Institute for Sports and Physical Activity (2003–2012), six community-based health-enhancing physical activity programmes targeting inactive, socially vulnerable groups were analysed, the programmes were initiated by different types of actors and used different strategies | **concept** program sustainability, not described in detail **assessment** realist synthesis to explore key combinations of contextual factors and mechanisms, longitudinal action research approach, data were collected between 2012 and 2014, interviews, time-line sessions, field visits, documentations of telephone calls and e-mail contacts  **sustained implementation**  the six programmes started between 2005 and 2014 and have been active since  **maintained PA effects**  N.A. |
| #10 | Lachance L, Quinn M and Kowalski-Dobson T^10^ | 2018/  USA | multiple case study, cross-sectional and longitudinal | community/  families and children | the "Food&Fitness" partnerships (F&F) were established to increase access to locally grown food and safe places for physical activity for children and families in communities with inequities across the United States, activities included changes in leadership, prioritising policy efforts and community action plans, funding from 2009-2016, racially, ethnically, and geographically diverse communities were selected, 9 communities started, 6 were funded through 2016 | **concept** working in partnerships beyond initial funding, outcomes to create lasting changes and increased capacity for new changes in the community **assessment** data were extracted for all F&F sites over the entire implementation period, quantitative and qualitative information on changes that occurred, evaluation tools were used cross-sectional and longitudinal (e.g. systems and policy change tracking forms, key informant interviews, grantee annual reports, community action plans)  **sustained implementation**  partnerships have created sustainable change through changes in infrastructures, systems, local policies, and practices  **maintained PA effects**  N.A. |
| #11 | Ochtera RD, Siemer CJ and Levine LT^11^ | 2018/  USA  (Colorado) | multiple case study | community/  whole-population | "LiveWell Colorado" has provided funding, oversight, and technical assistance from 2007 until 2016, the organisation providing "LiveWell" worked with selected communities to develop or strengthen existing HEAL (Healthy Eating and Active Living) efforts, including support for developing or strengthening community coalitions and funding for the community to hire a coordinator | **concept** sustained: continuation in part or in whole by entities and funding other than LiveWell; possibly sustainable: operation on reduced support and  funding by LiveWell; not sustained: strategies no longer supported by LiveWell and not continued by another community entity **assessment** evaluation data from 2016 of 8 communities included e.g. documentations, programme reports, check-ins, examination of strategies that were either sustained, possibly sustained or not sustained  **sustained implementation**  of 87 strategies, 69 (79%) have been sustained; 5 (6%) are in position to be sustained; and 13 (15%) were not sustained, policy and environment strategies were sustained more often compared with programme strategies despite being implemented less often  **maintained PA effects**  N.A. |
| #12 | Paez DC,  Reis RS,  Parra DC, Hoehner CM, Sarmiento OL, Barros M and Brownson RC^12^ | 2015/  Colombia, Brazil | two case  studies | community/ whole-population | 1) “Ciclovía Recreovía” and 2) “Academia da Cidade” provide opportunities to engage in regular exercise by offering e.g. free physical activity classes, the coordination of these programmes is imbedded within other local programmes, public partners are city departments, secretaries, and universities | **concept** maintenance: continuation of intervention effects on individuals and settings over time **assessment** RE-AIM framework to measure external validity, structured interviews with key informants  **sustained implementation**  1) Ciclovía Recreovía has been institutionalized since 1995 after modernisation; 2) Academia da Cidade has been institutionalized since 2002  **maintained PA effects**  N.A. |
| #13 | Quinn M, Kowalski-Dobson T and Lachance L^13^ | 2018/  USA | mixed-methods | community/ families and children | "Food and Fitness" partnerships see #10 | **concept** ability of the partnerships to focus their work, build individual and community capacity, execute the work, and produce systems and policy changes that would endure over time  **assessment** adapted Comprehensive Community Initiatives Sustainability (CCIS) Framework as online survey to assess individuals’ perceptions of their partnerships’ sustainability, key informant interviews  **sustained implementation**  core funding for the F& F partnerships ended in 2016, all partnerships are still together (in some form) and continuing  **maintained PA effects**  N.A. |
| #14 | Rütten A, Frahsa A, Rosenhäger N and Wolf A^14^ | 2015/  Germany | report | community/ women in difficult life situations | "BIG" see #8 | **concept** sustainable transfer: 1) "BIG" is transferred into the routines and social practices of the community, 2) it is transferred and applied to other contexts e.g. another community **assessment** multidimensional project evaluation  **sustained implementation**  example from one site: coordinator position initially located within the research team was transferred to the local sports department and funded by the city, PA offers have been continued  **maintained PA effects**  N.A. |

**REFERENCES**

1. van Acker R, De Bourdeaudhuij I, De Cocker K, et al. Sustainability of the whole-community project '10,000 Steps': a longitudinal study. *BMC Public Health* 2012; 12: 1–11.

2. Berg BK. Sustaining local physical activity programmes: lessons from the United States. *International Journal of Sport Policy and Politics* 2015; 8: 245–264.

3. Cheadle A, Egger R, LoGerfo JP, et al. Promoting sustainable community change in support of older adult physical activity: evaluation findings from the Southeast Seattle Senior Physical Activity Network (SESPAN). *Journal of Urban Health Bulletin of the New York Academy of Medicine* 2010; 87: 67–75.

4. Del Díaz Castillo A, Sarmiento OL, Reis RS, et al. Translating evidence to policy: urban interventions and physical activity promotion in Bogotá, Colombia and Curitiba, Brazil. *TBM* 2011; 1: 350–360.

5. Del Díaz Castillo A, González SA, Ríos AP, et al. Start small, dream big: experiences of physical activity in public spaces in Colombia. *Preventive Medicine* 2017; 103: S41-S50.

6. Draper CE, Kolbe-Alexander TL and Lambert EV. A retrospective evaluation of a community-based physical activity health promotion program. *Journal of Physical Activity and Health* 2009; 6: 578-588.

7. Haggis C, Sims-Gould J, Winters M, et al. Sustained impact of community-based physical activity interventions: key elements for success. *BMC Public Health* 2013; 13: 1–8.

8. Herbert-Maul A, Abu-Omar K, Frahsa A, et al. Transferring a community-based participatory research project to promote physical activity among socially disadvantaged women - experiences from 15 years of BIG. *Frontiers in Public Health* 2020; 8: 571413.

9. Herens M, Wagemakers A, Vaandrager L, et al. Contexts, mechanisms, and outcomes that matter in Dutch community-based physical activity programs targeting socially vulnerable groups. *Evaluation & the Health Professions* 2017; 40: 294–331.

10. Lachance L, Quinn M and Kowalski-Dobson T. Lessons learned from Food & Fitness about building successful partnerships: focus, capacity, and sustainability. *Health Promotion Practice* 2018; 19: 115S-124S.

11. Ochtera RD, Siemer CJ and Levine LT. Supporting community-based healthy eating and active living efforts in sustaining beyond the funding cycle. *American Journal of Preventive Medicine* 2018; 54: S133-S138.

12. Paez DC, Reis RS, Parra DC, et al. Bridging the gap between research and practice: an assessment of external validity of community-based physical activity programs in Bogotá, Colombia, and Recife, Brazil. *TBM* 2015; 5: 1–11.

13. Quinn M, Kowalski-Dobson T and Lachance L. Defining and measuring sustainability in the Food & Fitness Initiative. *Health Promotion Practice* 2018; 19: 78S-91S.

14. Rütten A, Frahsa A, Rosenhäger N, et al. Strukturelle Veränderung, Kontextualität und Transfer in der Gesundheitsförderung: die nachhaltige Implementierung des BIG-Projektes. *Gesundheitswesen* 2015; 77: S135-S136.
